# A Comparative Study to Determine the Effect of Intravenous Magnesium on Postoperative Bleeding after on Pump CABG in Patients Receiving Pre-Operative Aspirin

**Published:** 2009-04

**Authors:** Sampa Dutta Gupta, Soumendu Pal, Anupam Goswami, Sandip Bhadra, Sripurna Mandal, Chandashi Naskar, Sudakshina Mukherjee, Debasish Boral

**Affiliations:** 1 Assistant Professor, Department of Anaesthesioogy, N.B. Medical, College, West.Bengal; 2 RMO Cum Clinical Tutor, Department of Anaesthesioogy, N.B. Medical, College, West.Bengal; 3.Professor, Department of Anaesthesioogy, N.B. Medical, College, West.Bengal; 4 RMO Cum Clinical Tutor, Department of Anaesthesioogy, N.B. Medical, College, West.Bengal; 5 Student, Department of Anaesthesioogy. IPGME & R. Kolkata. 244, Acharyya, Jagadish Chandra Bose Road, Kolkata 700020; 6 Student, Department of Anaesthesioogy. IPGME & R. Kolkata. 244, Acharyya, Jagadish Chandra Bose Road, Kolkata 700020; 7 Associate Professor, Department of Anaesthesioogy. IPGME & R. Kolkata. 244, Acharyya, Jagadish Chandra Bose Road, Kolkata 700020; 8 House staff, Department of Anaesthesioogy. IPGME & R. Kolkata. 244, Acharyya, Jagadish Chandra Bose Road, Kolkata 700020

**Keywords:** Aspirin, Magnesium, CPB, CABG, Postoperative bleeding

## Abstract

**Summary:**

Hypomagnesaemia is a common complication after cardiopulmonary bypass (CPB) and predisposes to the development of cardiac arrhythmias. Previous studies showed that intravenous magnesium reduces the incidence of postoperative cardiac arrhythmias but it also inhibits platelet function. Our aim was to compare the postoperative blood loss in patients not receiving magnesium after CPB with the group who received magnesium and to compare the requirement of blood, fresh frozen plasma (FFP) and platelets within 24 hours after surgery. This prospective randomized controlled study was conducted in 80 adult patients on oral aspirin undergoing elective CABG requiring CPB. Group A patients had not received magnesium infusion after recovery from CPB. Group B patients received magnesium infusion after recovery from CPB. Postoperative bleeding was assessed in both the groups. All the data were statistically analyzed. There was a insignificant increase in 24 hours postoperative drainage in magnesium recipient group compared to control group (p>0.05). Requirements of blood and blood products to maintain haematocrit and coagulation profile revealed insignificant (p > 0.05). Increase in requirement of PRC, FFP and platelets in magnesium recipient patients than the control group. Incidence of atrial fibrillation (Gr A 2.5%, Gr B 2.5%) and atrial extrasystoles (Gr A 2.5%, Gr B 10%) revealed comparable (p > 0.05) between the groups, but incidence of ventricular arrhythmias were significantly (p<0.05) high in the patients of Gr A(17.5%) than Gr B(5%). To conclude, magnesium may be administered to patients who continue pre-operative aspirin to undergo on-pump CABG surgery.

## Introduction

Excessive post-cardiopulmonary bypass (CPB) bleeding is a major cause of post operative morbidity and mortality after cardiac surgery. The extracorporeal circulation leads to thrombocytopenia and platelet dysfunction^1^ which results in an increase in postoperative bleeding episodes, transfusion of blood and blood products and therefore increases the risk of transfusion related infections and immunological reactions.

Several studies have demonstrated the beneficial role of aspirin in patients of coronary artery diseases for reducing incidence of myocardial infarction^2^. By inhibiting platelet aggregation, aspirin reduces the incidence of infarction in post infarct patients and is also recommended for primary prevention of acute coronary syndromes. It also improves graft patency rates after coronary artery bypass grafting (CABG). Studies showed that, effects of magnesium impair platelet function, a 48% prolongation of bleeding time and a 40% inhibition of ADP induced platelet aggregation were observed in healthy volunteers after administration of 8 mM magnesium sulphate. Complete inhibition of platelet aggression at 10 mM magnesium were reported. By 8mM magnesium sulphate, serum magnesium concentration increases to 1.2 to 1.5 mM/L. Although data on the effect of magnesium on p-selectin expression and fibrinogen binding are limited.^3^ Paradoxically the mechanisms by which aspirin confers protection against myocardial infarction and graft closure after CABG may contribute to increased bleeding complications after cardiac surgery. There are several randomized clinical trials which showed that the incidence of bleeding complications after CABG is greater in patients who take aspirin before surgery^4^. So it has been customary to discontinue aspirin 7 – 10 days before surgery, which is the approximate duration of life of platelets. But recent studies have revealed that outcome is actually improved in patients who continue aspirin versus those who discontinue aspirin before cardiac surgery. Since the benefits of aspirin have been clearly demonstrated and it is not definitely associated with post - CPB bleeding, so it is reasonable to continue aspirin pre-operatively.

Hypomagnesaemia is common after cardiac surgery with an incidence of 70% after CPB due to haemodilution in the extracorporeal circulation, reduces the incidence of supraventricular and ventricular arrhythmias^5^. Hypomagnesaemia affects the cardiovascular system in a number of ways which include – coronary artery spasm, increased incidence of cardiac arrhythmias, digitalis related arrhythmias and has been associated with sudden death in patients with ischaemic heart disease. It also prolongs the duration of postoperative mechanical ventilatory support. Intravenous administration of magnesium reduces the incidence of postoperative ventricular and atrial arrhythmias^6^ and improves cardiac function after CABG. Magnesium is also effective in decreasing the number of episodes of postoperative atrial fibrillation^7^.

A controlled trial^8^ concluded that magnesium inhibited platelet function in vitro and in vivo. Although this antithrombotic effect of magnesium may be beneficial in patients after coronary revascularization, large dose magnesium therapy should be carefully considered in patients with impaired platelet function and coexisting bleeding disorders.

Whether magnesium exerts an additive inhibitory effect on platelet function in patients receiving aspirin preoperatively has not been investigated. We studied the effect of intravenous magnesium sulphate infusion on postoperative bleeding in patients of CABG on cardiopulmonary bypass who continued to take aspirin preoperatively.

Considering the results of previous studies, the aims of this study were to compare the postoperative blood loss in patients not receiving magnesium after CPB with the group who received magnesium and to compare the requirement of blood, fresh frozen plasma (FFP) and platelet within 24 hours after surgery.

## Methods

After approval of the Institutional Ethics Committee, this prospective, randomized, controlled, double blind study was conducted in the Department of Anaesthesiology, I.P.G.M.E&R / S.S.K.M. Hospital, Kolkata. After obtaining written, informed consent, eighty consecutive adult patients on oral aspirin undergoing elective CABG requiring CPB were prospectively randomized into two groups through a computer generated random number.

Group-A (Control Group) (n = 40)

Patients who did not receive magnesium infusion after recovery from CPB.

Group-B (Magnesium Group) (n = 40)

Patients who received magnesium infusion after recovering from CPB

Patients with history of recent thrombolytic therapy, warfarin therapy, history of previous cardiac surgery, hepatic disease, renal disease, hypersensitivity to study drugs, emergency patients who underwent re-exploration within the study period were excluded from the study.

All patients were premedicated with diazepam 0.1 mg.kg^−1^ orally and their usual dose of aspirin, beta blocker and calcium channel blocker in the morning. In the operation theatre, invasive blood pressure, pulse oximetry, central venous pressure, temperature, ECG and arterial blood gases, serum electrolytes, (Na^+^.K^+^.Ca^++^) were routinely monitored in all patients. After proper preoxygenation, all patients were induced with fentanyl 10 mcg.kg^−1^ iv and midazolam 0.1 to 0.4 mg.kg^−1^ iv titrated to the effect, tracheal intubation were facilitated with pancuronium 0.1 mg.kg^−1^ iv. Anaesthesia were maintained with oxygen in air 40%: 60% along with isoflurane, intermittent bolus of fentanyl and vecuronium along with infusion of propofol at 50 to 100mg.kg^−1^.min^−1^. The extracorporeal circulation circuit was primed with 1800 ml of Ringer's lactate and 100 ml 20% human albumin. A capillary membrane oxygenator, arterial line filter, polyvinyl chloride tubings for the pump were used in all patients. Patients were anticoagulated with heparin 3 mg.kg^−1^ iv and repeated as necessary to maintain activated clotting time (ACT)>400 secs. Moderate systemic hypothermia, non pulsatile, filtered, arterial flow and gravity venous drainage were common features of the operation. All patients received epsilon aminocaproic acid (EACA) 100 mg.kg^−1^ iv bolus and 10 mg.kg^−1^.hr^−1^ for 5 hours after administration of heparin. At the end of the operation, protamine sulphate was used to neutralize heparin in the ratio of 1.3 mg protamine per mg of heparin.

At the end of CPB, Group A (Control Group) received 100 ml normal saline iv and Group B (Magnesium Group) received 2 gm magnesium sulphate in 100 mL 0.9% NaCl solution (12.5 mL/h) just after cardiopulmonary bypass with the opening of cross clamp and continued for 72 hours postoperative period. The control group received only 100 mL 0.9% NaCl solution (12.5 mL/h) at the same time points. Infusions were guided by and directed to maintain normal tissue perfusion as indicated by normal urine output, absence of metabolic acidosis. Potassium (20mEq potassium chloride in 100 mL normal saline solution) was administered as required to maintain the concentration of potassium at 4 mmol/L. Inotropic agents were not used to raise perfusion pressure during cardiopulmonary bypass. However, pump flow was never allowed to fall to less than 2.2 L/m^2^. In addition to potassium and calcium measurements obtained during operation and in the intensive care unit. A blood gas analysis was also performed at the latter time points and oxygen saturation obtained by pulse oximetry.

Postoperative monitoring included invasive blood pressure, pulse oximetry, central venous pressure, temperature, ECG and arterial blood gases, serum electrolytes,(Na^+^.K^+^.Ca^++^), urine output, volume of mediastinal drainage and other bleeding (if any).

Homologous blood was transfused when hemoglobin values dropped below 8.5 gm%. The amount of mediastinal drainage was noted for 24 hours. Perioperative transfusion therapy was guided with routine coagulation monitoring. Platelet concentrate was transfused if ACT and PT were not prolonged but patient had persistent bleeding (> 150 ml/hr for > 2 hrs). FFP was transfused if PT was prolonged by >50%.

Statistical analysis: Sample size was calculated from a previous study^9^ on the basis of the anticipated difference in 24hrs mean expected blood loss between the two groups. Assuming that the mean expected blood loss in 24 hrs would be 500 ml in the control group with a standard deviation of 300 ml, it was calculated that 40 patients per group would be required to detect a significant difference with 80% power and 5% probability of Type I error.

Numerical Parametric data was given as Mean±SD. Ordinal and nominal data was expressed in cross table as number (%) of patients in each ordinal and nominal category. Numerical parametric data was compared by unpaired t – test (independent sample t – test). Categorical data was compared by Chi-square test / Fischer's exact test *p*<0.05 was considered statistically significant.

## Results

Table 1 shows that, the demographic profile of the two groups were comparable, (p>0.05) with respect to age & sex and the difference was not statistically significant

**Table 1:** Demographic profile

Group	Mean Age(yrs)	Age (yrs) Range	Sex (M / F)
A(n=40)	62 ± 8	55 - 69	13/2
B(n=40)	62 ± 12	48 - 72	12/3
			
p values	(p>0.05)		

Table 2 shows that haematologic variable of the two groups was comparable and the difference was not statistically significant.

**Table 2:** Preoperative haematologic values & coagulation times(mean±SD)

Haematologic variables	Group A (n=40)	Group B (n=40)
RBC (×10^12^/L)	4.72 ± 0.44	4.81 ± 0.32
Haematocrit	36.0 ± 6.6	34.8 ± 5.8
PT (Secs.)*	0.95 ± 0.29	0.93 ± 0.38
APTT (Secs.)	30 ± 5.4	30.4 ± 3.1
Platelets (× 10^9^/L)	213 ± 63	212 ± 43
Serum Magnesium (mM/L)	0.83±0.12	0.81±0.1
		
p values	(p>0.05)	

RBC-Red blood cell. PT- Prothrombin time, APTT – Activated partial thromboplastin time

* PT expressed as a fraction of normal values

Table 3 shows that, the distribution of risk factors and associated diseases like obesity, congestive heart failure, history of smoking, diabetes mellitus and hypertension was comparable between the two groups.

**Table 3:** Risk factors & associated diseases

Risk factors	Group A (n=40)	Group B (n=40)
*BMI > 25 kg/M^2^(%)	12.7	14.9
Preoperative**CHF (%)	11.6	10.8
H/O smoking (%)	52.9	53.1
Diabetes Mellitus (%)	26.5	24.4
Hypertension (%)	90	92
		
p value	(p>0.05)	

* BMI – Body mass index.

** Congestive heart failure

Table 4 shows that, the data from the intraoperative observations revealed no statistically significant difference in the percentage of left internal mammary artery (LIMA) harvesting, number of vessels bypassed, CPB time, total dose of heparin and protamine, pre-operative and postoperative haematocrit values.

**Table 4:** Intra- operative observations

Variabes	Group A (n=40)	Group B (n=40)
*LIMA graft (%)	78.2	72.4
No. of grafts	3.3±0.6	3.5±0.8
**CPB time (min.)	136±53	134±68
Heparin (mg)	157.5±18.4	147±17.3
Protamine (mg)	210±20.4	200±21.5
Preoperative haematocrit(%)	36.0±6.6	34.8±5.8
Postoperative haematocrit(%)	38.7±2.0	38.4±1.8
		
		
p value	(p>0.05)	

* LIMA – Left internal mammary artery

** Cardiopulmonary bypass.

Table 5 shows that, the incidence of cardiac arrhythmias after CPB were compared and revealed that, the incidence of atrial arrhythmias were comparable between the two groups. A significant increase in the incidence of ventricular arrhythmias in Group A(17.5%) was observed against Group B (5%) (p<0.05). Out of the 7 patients suffered ventricular tachycardia of Group A (Control Group), 4 patients responded to treatment with DC Cardioversion and amiodarone iv, while 3 patients suffered ventricular extrasystoles were haemodynamically insignificant treated with iv amiodarone. Only 2 patients in Group B (Magnesium Group) suffered ventricular extrasystoles which did not require any treatment.

**Table 5:** Incidence of cardiac arrhythmias(n)

Cardiac arrhythmias	Group A (n=40)	Group B (n=40)	P Value
Atrial Fibrillation	1(2.5%)	1(2.5%)	>0.05
Atrial extrasystoles	5(12.5%)	4(10%)	>0.05
Ventricular arrhythmias	7(17.5%)	2(5%)	>0.05

Table 6 shows that, total collection of mediastinal drainage in 24 hours postoperative period were compared and found that there was a modest increase in drainage in Magnesium Group compared to Control Group but it was statistically not significant.

**Table 6:** Volume of mediastinal drainage in 24 hours postoperative period

Volume of mediastinal drainage	Group A (n=40)	Group B (n=40)	P Value
Total (ml)	529±388	560±465	(p>0.05)
>1000 ml (%)	5 (12.5%)	8 (20%)	>0.05)
> 1500 ml (%)	1 (2.50%)	2 (5.%)	>0.05)

Table 7 shows that, when the requirement of blood and blood products to maintain haematocrit and coagulation profile was compared between the groups and there was increased requirement of packed RBC(PRC), FFP and platelets in Group B (Magnesium Group) but was not statistically significant.

**Table 7:** Transfusion requirements of allogenic blood products[mean±SD(Range)]

	Group A(n=40) Mean±SD	Group A(n=40) Mean±SD
Packed Red cells.(PRC)	1.3±2.0(0-16)	1.4±1.9 (0 - 10)
Fresh Frozen Plasma.(FFP)	0.4±1.1(0 - 8)	0.5±1.3 (0 - 10)
Platelets	0.5±1.1(0- 6)	0.5±1.2 (0 - 6)
		
		
p value	(p>0.05)	

Other relevant data revealed no significant differences in requirement of inotropes and vasodilators between the two groups during the study period. Urine output were comparable between the two groups,. Patients needed re-exploration were not included in the study. Total five patients expired (three from Group A and two from Group B) and were excluded from this study.

## Discussion

Aspirin exerts its antithrombotic and antiplatelet effects by irreversibly acetylating a serine residue, thus inhibiting the cyclooxygenase and hydroperoxidase reactions necessary for the production of thromboxane A_2_, a potent vasoconstrictor and platelet aggregator^10^. While perioperative aspirin use can reduce the incidence of coronary artery thrombosis, and improve graft patency after CABG. A previous study indicated that aspirin increased postoperative bleeding and transfusion requirements after cardiac surgery^11^. But another study had not confirmed this effect on haemostasis, instead it has been revealed that outcome is actually improved in patients who continue aspirin in the perioperative period when compared to those patients who discontinue aspirin^12^.

After CPB, hypomagnesaemia is common. Patients undergoing cardiac surgery are at risk of magnesium deficiency due to haemodilution in the extracorporeal circuit, diuretic therapy and heart failure^13^. Haemodilution at the start of CPB causes 17% decrease in plasma magnesium which persists until the first post operative day. Also, assessment of magnesium status is a complex area. Only 0.3% total body magnesium is found in serum and samples are affected by magnesium of red blood cells. Measurements of ionized magnesium is prone to interference from other cations like Ca^++^. Thus, although monitoring of serum Mg^++^ has a role in guiding magnesium therapy in acute situations like established cardiac arrhythmias and hypomagnesaemic convulsions; we have measured baseline serum electrolytes levels including serum magnesium but due to logistic difficulties serum magnesium level could not be measured during intraoperative and postoperative period in all patients. This is because hypomagnesaemia is common during the immediate post CPB period. We have used magnesium only in patients who had adequate urine output. All our study patients had adequate urine output and were haemodynamically stable during the study period.

Postoperative hypomagnesaemia is associated with prolonged ventilatory support and an increased incidence of cardiac arrhythmias, which may lead to prolonged PR intervals, wide QRS complexes, depressed ST segments, inverted T waves and prolonged QT intervals^14^. The administration of magnesium after CPB reduces the incidence of supraventricular and ventricular arrhythmias and increases cardiac index and left ventricular stroke work index^15^. Previous study showed that, the incidence of ventricular arrhythmias in patients not received magnesium for 24hrs post CPB period was nearly three times that found in the magnesium treated group^16^. Present study studied the incidence of arrhythmias from the immediate post CPB period and continued upto 24 hrs postoperative period in ICU. Here the incidence of atrial arrhythmias was similar between Group A (Control Group): 25% and Group B (Magnesium Group): 20%. But the incidence of ventricular arrhythmias was 35% in Group A (Control Group) compared to 10% in Group B (Magnesium Group). DC cardioversion and inj. amiodarone were instituted for the management of significant ventricular arrhythmias. Thus hypomagnesaemia may be a significant contributory factor that predispose towards the development of postoperative arrhythmias and the intraoperative administration of magnesium may reduce the incidence of serious ventricular arrhythmias following coronary revascularization.

Magnesium can lead to significant dose dependent prolongation of bleeding time, inhibition of platelet aggregation, P – selectin expression and fibrinogen binding to the platelet GP IIb/IIIa receptor in patients undergoing cardiac surgery. The results of the present study show that Group B (Magnesium Group) patients had a modest increase in the volume of mediastinal drainage noted in the 24 hours postoperative period and homologous blood requirements were also greater than Group A (Control Group) patients, but the difference was not statistically significant. Preoperative platelet function was evaluated by platelet count, prothrombin time and activated clotting time.

Blood-saving techniques are of great interest to cardiac surgeons due to the risk of allergic reactions and viral infections. Intra-operative antifibrinolytic therapy is an effective means of reducing postoperative bleeding after CPB^17^. Epsilon aminocaproic acid (EACA) an antifibrinolytic drug, prevents fibrinolysis and platelet activation during CPB. It also prevents activation of plasminogen. EACA binds to lysine binding sites on plasminogen and fibrinogen and thereby inhibits plasminogen activator and plasmin release. When administered before CPB it inhibits fibrinolysis, decreases mediastinal bleeding and decreases transfusion requirements^18^. In the present study both Group A (Control Group) and Group B (Magnesium Group) patients have received equivalent dosages of intravenous EACA infusion. So EACA should not have influenced the results of the study as its effect on post operative bleeding was equal in both the groups of patients.

Thus, intravenous magnesium infusion (2 mg Magnesium Sulphate in 100 ml normal saline) may be administered to patients posted for CABG with CPB who receive perioperative aspirin therapy without any significant increase in post operative bleeding and blood or blood product requirements. It may be concluded from the study that, magnesium may be used for its beneficial effect of preventing cardiac arrhythmias in the post-CPB period, without significant increase in risk of peri-operative haemorrhage and blood product requirements in on pump CABG patients who continue pre-operative aspirin.
